# Comprehensive 4E (energy, exergy, economic, and environmental) assessment of a repowered natural gas-fired combined power plant

**DOI:** 10.1038/s41598-026-37499-7

**Published:** 2026-01-28

**Authors:** T. E. Boukelia, A. Bouhala, Y. Cheurfi, R. Bessaïh, A. Tanougast, K. Hriczó

**Affiliations:** 1 Mechanical Engineering Laboratory, Faculty of Science and Technology, Mohamed Seddik Benyahia University, Jijel, Algeria; 2 Mechanical and Advanced Materials Laboratory, Polytechnic School ofConstantine, Constantine, Algeria; 3 LEAP Laboratory, Department of Mechanical Engineering, FrèresMentouri University-Constantine, 1, Route de Ain El. Bey, Constantine, Algeria; 4 Institute of Mathematics, Faculty of Mechanical Engineering andInformatics, University of Miskolc, Miskolc Egyetemvaros, Miskolc, 3515 Hungary

**Keywords:** 4E study, Natural gas-fired combined power plant, Configuration, Modeling, Regeneration., Energy science and technology, Engineering, Environmental sciences

## Abstract

Natural gas-fired combined power plants (NGFCPP) stand out as a promising technology for electricity generation, boasting high conversion efficiency and relatively low carbon dioxide emissions. Numerous researchers have explored diverse strategies to further optimize the performances of these systems. The main aims of this work are to model the 4E (energy, exergy, economic, and environmental) performances of a new design of an NGFCPP and compare them to those of an operating conventional plant (Hadjret Enouss plant). The obtained results show that the plant operating with the new design achieved a higher energy efficiency of 63.77%, compared to the Hadjret Enouss plant’s 58.87%. Moreover, its exergy efficiency of 56.58% also surpassed the Hadjret Enouss plant’s 55.54%. Although the NPV of the new design was slightly lower at 764.57 M€ compared to the Hadjret Enouss plant’s 776 M€, the developed plant demonstrated superior sustainability with the lowest CO_2_ emissions at 40.77 kg/s and the least cooling water consumption at 5.984 m³/s. In conclusion, this new design offers significant long-term benefits, with the potential to save a considerable amount of fuel and reduce environmental impact over its lifetime. For example, over 35 years of operation, the developed plant can save 154.526 million kg of natural gas compared to conventional NGFCPPs, leading to an annual reduction in CO_2_ emissions of approximately 24.28 million kg.

## Introduction

Energy and climate change are the two major concerns of this century. Carbon dioxide (CO_2_) is considered the main cause of global warming, with over 40% of CO_2_ emissions originating from the power industry. According to the International Energy Agency (IEA), electricity demand is expected to increase by about 80% from 2012 to 2040^1^. As a result, significant efforts are underway to produce clean, sustainable, and efficient electric power.

Natural gas-fired combined power plants (NGFCPPs) present a promising solution to produce electricity due to their high conversion efficiency and low CO_2_ emissions. In Algeria as an example, electricity is primarily generated from natural gas. The national power production infrastructure is dominated by eight NGFCPPs with a total capacity of 10,023 MW, representing 43.58% of the national electricity production^2^. These power plants have undergone remarkable developments to improve their energy and exergy performances, economic viability, and to reduce CO_2_ emissions. Various methods have been employed by numerous researchers to enhance the performances of these systems, including (i) improving operational factors and conditions (compression ratio, combustion temperature, condenser pressure, etc.) that significantly impact the performances of gas and steam turbines. (ii) Minimizing friction and irreversibilities by increasing efficiencies (isentropic, mechanical, etc.). (iii) Making modifications to the configuration and thermodynamic cycle of the base cycle (regeneration, reheating, etc.).

Regarding the third research direction, many works and studies have been presented in the recent years to investigate such subject. At instance, Sen et al.^3^ conducted a study on the effect of ambient temperature on electricity production in a NGFCPP. In this regard, they examined the changes in power generation of a typical 240 MW NGFCPP based on ambient temperature from real data recorded over approximately fourteen years in İzmir, Turkey. Their results confirmed that increasing the ambient temperature leads to a decrease in the dispatch capacity of the gas turbine block, thus, both the efficiency and the total power output of the installation decreased. While Almansoori and Dadach^4^ simulated the performances of a natural gas combined cycle power plant with an electricity production of 620 MW using Aspen Hysys software based on the Soave-Redlich-Kwong equation of state. The energy, exergy, environmental performance metrics were considered in the simulations. They identified the combustor as the dominant source of irreversibility and environmental burden, accounting for 24.35% of total exergy destruction with the lowest exergy efficiency (75.65%) and the highest environmental contribution (32.19%). On the other hand, Aliyu et al.^5^ conducted an energy, exergy, and parametric analysis of a triple pressure NGFCPP. The obtained results reveals that the combustor followed by the evaporator exhibit the highest irreversibility due to the large temperature gradients in these two components, while the turbine achieving the highest exergy efficiency (more than 92%). Moreover, Lee et al.^6^ developed and validated a simulation program for predicting the electricity production capacity of a combined power plant. In this regard, different curves and data based on real operation of the investigated plant were employed. The first step in their work was the modelling of the system under design and off-design operating based on respective seven-day periods. While the second step was establishing a model to predict the power generation of this installation in the past and future. An error of less than 2% between the data generated by the tool and those of real operating data. Almutairi et al.^7^ conducted an energy and exergy analysis of a combined cycle power plant (in Sabiya, Kuwait). The installation employs an advanced triple-pressure reheat Heat Recovery Steam Generator (HRSG), and its performance was examined under various operating conditions. The proposed system was modeled using IPSEpro software and validated with the manufacturer data. The results confirmed that the combustion chamber is consistently the primary source of exergy destruction, accounting for approximately 61%, followed by the HRSG. Based on the assumption of the limitations of analysis and optimization focused solely on the techno-economic performances of a combined cycle gas turbine (CCGT), Chen et al.^8^ proposed an innovative methodology to optimize a CCGT. Their approach included a decision-making algorithm supported by different scenarios, such as ambient climatic conditions, electricity demand and price, and plant degradation. This methodology was applied to an existing plant operating in the UK. Based on the developed approach, the authors recommended to ensure the operation of the power plant at reduced outputs when possible, to maintain component integrity, thereby extending the plant’s service life. Contrarily, Xu et al.^9^ compared the economic performances of replacing the steam Rankine block with a triple-compression sCO_2_ power cycle in a coal-fired power plant. The system considered multiple thermal enhancements, including intercooling and reheating, and resistant materials were selected to ensure the plant’s safety. Moreover, the authors incorporated a detailed analysis of the sCO_2_ boiler and recuperator within their developed economic model. Their results indicate that the proposed sCO_2_ layout enhances the yield of the installation to over 49%, compared to less than 48.2% for a conventional steam Rankine cycle. Furthermore, the Levelized Cost of Electricity decreased by 1.32% for the first block relative to the second one. While Nikbakht Naserabad et al.^10^ based their study on the thermodynamics performances, particularly exergy ones, of an updated 320 MW old steam power plant. Different repowering scenarios were considered, featuring three different flow rates and two gas turbines designs, along with several enhancements introduced in the steam block. has also been analyzed. By considering this repowering scenario, the repowered configuration reaches a peak thermal efficiency exceeding 52%. Another repowering work was presented by Adeli et al.^11^, where they focused their analysis on the feed-water-heating process, using the typical 320 MW Isfahan power plant as a case study. They replaced either the low-pressure or high-pressure heat exchangers by new gas-water heat exchanger. Moreover, different gas turbine designs were explored. In this regard, exergy and exergy dimensions of these proposed layouts were investigated in this work. While Otitoju et al.^12^ aimed to reduce CO_2_ emissions by integrating post-combustion carbon capture technology into a NGFCPP. They assessed the techno-economic performances of the installation, which had a net output of 250 MW, using Aspen Plus software. Three different layouts were discussed and compared. Another environmental metric in NGFCPP considered by Nakamura et al.^13^ in their simulation work was the emission of NOx during varied operational loads of this type of plants when integrating a monolithic selective catalytic reduction system. Simulations were developed and validated to model the modified de-NOx reaction scheme under diverse NGFCPP exhaust gas compositions, encompassing both steady-state and transient operating conditions. They assessed the robustness of de-NOX performance under operational fluctuations associated with high renewable energy penetration, using validated simulations of a modified reaction scheme for a selective catalytic reduction under steady and transient NGCC exhaust conditions. Moreover, Abdollahian and Ameri^14^ analyzed the effect of incorporating a supplementary firing process in a NGFCPP on its energy and exergy performances. Their main goal was to boost the generated power by these installations, particularly in response to varied loads in grid electricity demand. To achieve this goal, they considered three different modes of operation from both energy and exergy perspectives. They found that the plant’s operation under part-load conditions resulted in higher exergy destruction. Méndez-Cruz et al.^15^ performed a comprehensive investigation of an operating hybrid combined power plant installed in the Valley of Mexico, considering both full and part-load conditions. The study evaluated several parameters, including power generation, energy efficiency, and fuel consumption. Their results indicated that under full-load operation, and by implementing an enhanced Heat Recovery Steam Generator, the proposed plant saved approximately 33,903.36 tons of fuel annually and increased the exergy efficiency to 54.08%. Keying et al.^16^ proposed to combine an exhaust gas recirculation control with variable inlet guide vanes and fuel flow controls to improve the performances of a CCGT under part-load operations. In this regard, they developed and validated design and off-design models to investigate the performances of these plants. The obtained results show that the thermal efficiency of the studied layout was enhanced across various operating loads, including those below 50%. Another analysis of the part-load operation of a CCGT was performed by the same authors^17^, where they presented a detailed energy and exergy analysis of the installation proposed in their previous work, including the topping, bottoming, and combined cycles. They found that the efficiency of the entire installation was increased by 0.97–1.21% points across a wide operating range (40–90% loads). While Esmaeilion and Soltani^18^ raised the dispatch capacity of a combined power plant by incorporating an energy storage system with a thermal reservoir. In this layout, they investigated its polygeneration capabilities where the installation is capable of producing electrical power and freshwater. Moreover, an optimization approach based on the Grey Wolf algorithm was applied to enhance its performances. On the other hand, different artificial intelligence algorithms have been applied by several researchers, including machine learning techniques^19^, artificial neural network (ANN) models^20,21^, hybrid ANNs optimized by the Electrostatic Discharge Algorithm^22^, as well as multi-objective optimization^23^, and Adaptive Neuro-Fuzzy Inference Systems utilized to predict and enhance the performances of this type of large-scale power generation plants.

Although numerous studies have investigated the development and performance assessment of natural gas-fired combined cycle power plants (NGFCPPs)^24–31^, most of them are limited to conventional configurations and focus on partial evaluations, such as energy and/or exergy and/or economic analyses alone. In contrast, the present study provides, for the first time, a comprehensive 4E assessment (energy, exergy, economic, and environmental) of an updated NGFCPP design incorporating combined thermodynamic enhancements including reheating, regeneration, and heating, to enable full repowering. Unlike existing works, the proposed configuration is systematically compared with an operating conventional NGFCPP, allowing a clear quantification of the thermodynamic, economic, and environmental gains achieved through the proposed modifications. This integrated approach not only deepens the understanding of irreversibility reduction and efficiency improvement mechanisms but also provides practical insights for repowering strategies in modern combined-cycle power plants. Thus, the main objective of the present study is to develop and evaluate an updated NGFCPP incorporating thermodynamic enhancements, in order to achieve full repowering and improved overall performance. A comprehensive energy, exergy, economic, and environmental (4E) assessment is conducted to quantify the impacts of such modifications. The analysis is presented in terms of energy and exergy efficiencies, energy losses, and exergy destruction, as well as key economic indicators including total investment cost and net present value. In addition, the environmental performance is evaluated through CO₂ emissions and water consumption associated with the cooling process. The results are systematically compared with those of an existing conventional NGFCPP to highlight the benefits and trade-offs of the proposed design.

## Data and methodology

In the present study, a novel design of a NGFCPP is proposed, and its 4E performances will be presented and analyzed. To achieve this goal, a well-defined four-step methodology will be followed:

1. First, the main data and inputs of the investigated plant will be collected. These include technical data related to the thermodynamic model, such as the operating temperatures and pressures at the main state points, the isentropic and mechanical efficiencies of the turbomachinery (compressors, gas turbines, steam turbines, and pumps), and the performance parameters of the heat exchangers, including the heat recovery steam generator (HRSG), feedwater heaters, condenser, reheater, and economizer. In addition, economic data required for the techno-economic analysis are gathered, including cost correlations used to estimate the initial investment costs of each major component of the installation, as well as parameters related to operation and maintenance costs, fuel cost, and plant lifetime assumptions.
2. The mathematical models, which consider the thermodynamic, economic, and environmental performances, will be described and validated. The energy and exergy models are based on mass and energy conservation and the second law of thermodynamics. The thermodynamic model is validated using manufacturer and operational data from existing power plants (Hadjret Enouss and Achouat). The economic model employs cost correlations from the literature to estimate the initial investment cost and equations to evaluate the operation and maintenance costs. Finally, the environmental assessment considers CO₂ emissions based on the stoichiometric combustion of natural gas and water consumption estimated using a thermodynamic approach.
3. These performances will be compared to those of the conventional design of most operating NGFCPPs, as well as those of an existing steam power plant. n particular, the 4E performances are compared with operational data from the Hadjret Enouss combined cycle power plant and the Achouat steam power plant.
4. Finally, the details of these 4E performances of the updated plant will be presented and discussed.

### Studied configurations

As mentioned before, a novel design of a NGFCPP will be proposed, and its 4E performances will be evaluated and compared to those of a conventional design, as well as of an operating steam turbine power plant. The conventional design of the studied plant is similar to that of Hadjret Enouss power plant in Tipaza, Algeria. This power plant has a net power output of approximately 1227 MW (guaranteed at an ambient temperature of 25 °C using natural gas as the primary fuel). The Hadjret Enouss power plant consists of three units, each providing a net power output of approximately 407 MW. On the other hand, the modified design with the same capacity is based on the combination of the 9FB gas turbine model with a regenerative steam turbine (ST) block, which includes seven feedwater heaters (FWH) and a deaerator. This arrangement is similar to the existing design of most operating steam turbines, with the Achouat power plant in Jijel, Algeria being taken as the reference plant for the steam turbine power plant in the present study.

**Fig. 1:**
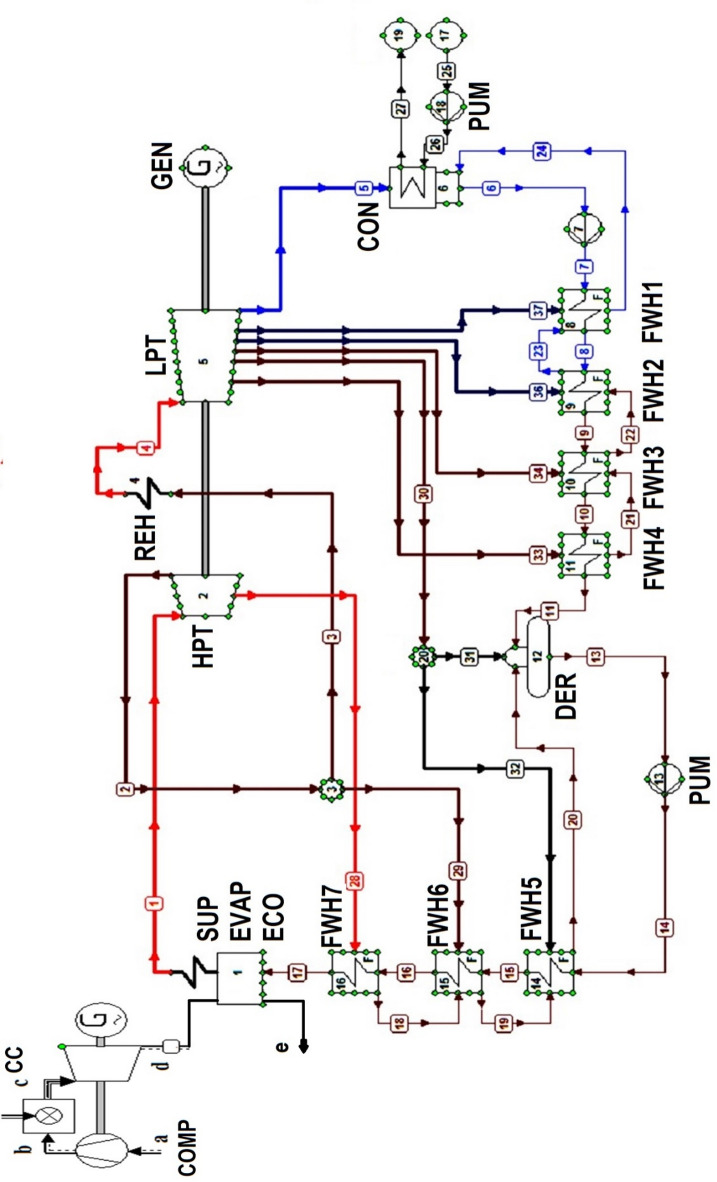
Studied configuration.

In this developed layout, ambient air is drawn in and compressed by a compressor driven by the turbine. The compressed air then moves to the combustion chamber where it is burned (with natural gas) at a high temperature of approximately 1396 °C. These hot gases expand through different stages of the gas turbine, producing mechanical work, which is then converted to electrical power by the generator. On the other side, extraction pumps feed water to the low-pressure FWHs, which heat the water using steam extracted from the low-pressure turbine (LPT). The water then enters the deaerator (mixing preheater) to remove O_2_ and other dissolved gases in the water that cause severe corrosion in the installation. It is then pumped to the high-pressure FWHs [heated by steam extracted from the high-pressure turbine (HPT)] using feedwater pumps. After that, the water is circulated through the heat recovery steam generator (HRSG), changing its phase to superheated steam using the heat recovered from the exhaust gases of the gas turbine. The steam is then expanded in the HPT. However, not all of its thermal energy is transferred in this equipment, so it is sent back to the reheater to be reheated. Following reheating, the steam passes through the LPT. At the end of the expansion, the steam is sent to a condenser where heat exchange between the steam and cold water leads to the condensation of the hot steam, and the cycle is repeated. The schematic of this proposed layout is shown in Fig. 1, while the data and inputs used for the modeling and simulation of the considered layout with its two blocks are presented in Tables 1 and 2, and 3 for the gas turbine block, steam turbine block, and FWHs, respectively.

**Table 1:** Manufacturer data for the 9FB GT block^32,33^.

Parameter	Value
Compressor inlet temperature Ta (°C)	25
Compressor inlet pressure Pa (bar)	0.9987
Compression ratio r_p_ (–)	18.3
Lower heating value LHV (kJ/kg)	45,720
Air mass flow rate (kg/s)	603.33
Combustion temperature (°C)	1669
Flue gas outlet temperature (°C)	642.8
Compressor isentropic efficiency (%)	89
Compressor mechanical efficiency (%)	97
Combustion efficiency (%)	94.04
Pressure drop in combustion chamber (%)	4.00
Turbine isentropic efficiency (%)	90.1
Turbine mechanical efficiency (%)	98
Generator efficiency (%)	95

**Table 2:** Thermodynamic data for key parameters in the studied configuration (ST block)^32,33^.

Parameter	Value
Ambient environmental conditions	
Temperature (°C)	25
Pressure (bar)	1.013
Conditions at the inlet of the HPT	
Temperature (°C)	565.1
Pressure (bar)	120.8
Conditions at the outlet of the HPT	
Pressure (bar)	25.20
Conditions at the inlet of the LPT	
Temperature (°C)	565
Pressure (bar)	22.85
Conditions de the outlet of the LPT	
Pressure (bar)	0.0496
HPT Isentropic efficiency (%)	88
LPT Isentropic efficiency (%)	88
Mechanical efficiency of HPT/LBP (%)	97.5
Isentropic efficiency of Pumps (%)	87
Regenerator efficiency (%)	99
Pressure Drop ΔP in boiler (bar)	34.6
Pressure Drop ΔP in reheater (bar)	3.22
Pressure Drop ΔP in deaerator (bar)	0.6
Pressure at the deaerator outlet (bar)	7.611
Condensation pressure (bar)	0.04958
Temperature at the reheater outlet (°C)	565

**Table 3:** Assumed data of the FWHs.

	FWH 1	FWH 2	FWH 3	FWH 4	FWH 5	FWH 6	FWH 7
$\:{\varDelta\:\boldsymbol{P}}_{1}$(bar)	0.3	0.6	0.3	0.6	2.4	2.4	2.3
$\:{\boldsymbol{P}}_{\boldsymbol{i}\boldsymbol{n}.2}$(bar)	0.241	1.17	2.54	5.79	10.9	26.7	35.2
$\:{\varDelta\:\boldsymbol{P}}_{2}$(bar)	0	0	0	0	0	0	0
$\:{\varDelta\:\boldsymbol{T}}_{\boldsymbol{L}}$ (°C)	5	5	5	5	7	7	7
$\:{\varDelta\:\boldsymbol{T}}_{\boldsymbol{H}}$ (°C)	3	3	3	3	0	0	0

### Considered assumptions

The thermodynamic modeling of the studied layout can be achieved by combining the analysis of each subsystem. Therefore, introducing some assumptions proves crucial for implementing the mathematical models. The following assumptions are made in the present study:

- The plant has a net capacity of 407 MW at an ambient temperature of 25 °C.
- Each component of the system is considered a control volume with steady-state flow.
- Kinetic and potential energies are neglected.
- The temperature and pressure of the air at the compressor inlet are the ambient temperature and atmospheric pressure, respectively.
- Refprop thermodynamic tables are used to extract the thermo-physical properties and state variables of the working fluids (air and steam).
- Water is in a saturated liquid state at the outlet of the condenser and preheater. It exits the evaporator as saturated vapor.

Furthermore, during our environmental analysis, the following points are taken into account:

- The combustion reaction is assumed to be stoichiometric.
- The fuel used is natural gas (methane CH_4_).
- Air is composed of 20% oxygen (O_2_) and 80% nitrogen (N_2_).
- CO_2_ is considered the only gas emitted by the studied power plants.

### Mathematical modelling

#### Thermodynamic modelling

In this subsection, the mathematical model for simulating the thermodynamic performances of this NGFCPP’s design is presented. Initially, energy and exergy models based on the first and second laws of thermodynamics, respectively, are provided for each component of the system. Moreover, to estimate the thermophysical properties and state variables of the working fluids (water/steam and air/flue gases) at each point of the power generation process, such as temperature, pressure, enthalpy, entropy, density, etc., REFPROP is utilized.

The energy analysis uses the conservation of mass and energy principles (the first law of thermodynamics). Applying these conservation laws to the individual components of the system, the following mass and energy balance equations are employed:

1 $$\:\sum\:{\dot{m}}_{in}=\sum\:{\dot{m}}_{out}$$

2 $$\:\dot{Q}+\sum\:{\dot{m}}_{in}{h}_{in}=\dot{W}+\sum\:{\dot{m}}_{out}{h}_{out}$$

On the other hand, Exergy, representing the maximum useful work extractable from a system in a given state, considers both the quantity of energy and its quality (i.e., work potential or usefulness). Exergy is divided into four parts: physical, chemical, kinetic, and potential. In the present study, and according to the considered assumptions, the variations in kinetic and potential exergy are considered negligible.

The exergy analysis modeling is defined based on the second law of thermodynamics. The results of this analysis can be used to identify locations of irreversibility, and propose various methods to reduce these irreversibilities to the lowest possible value by improving the system’s performances. Thus, the total exergy of each component is the sum of the physical and chemical exergy components:

3 $$\:\dot{E}x=\:{\dot{E}x}_{chm}+{\dot{E}x}_{ph}$$

where the physical component is related to the difference in physical state, in terms of temperature and pressure, between this substance and the reference environment:

4 $$\:{\dot{E}x}_{ph}=\:\dot{m}\left(h-{h}_{0}\right)-{T}_{0}\left(s-{s}_{0}\right)$$

While the chemical exergy is linked to the difference in chemical composition of this substance compared to the environment, at the temperature and pressure conditions of the environment, and expressed by:

5 $$\:{ex}^{chm}=\:\sum\:_{i=1}^{n}{R}_{i}{T}_{0}\:ln\left(\frac{{y}_{i}}{{y}_{i}^{0}}\right)$$

Here, where $\:{R}_{i}$ is the gas constant of component i, $\:{T}_{0}$ is the reference temperature, $\:{y}_{i}$ is the mole fraction of component i in the substance, and $\:{y}_{i}^{0}$ is the mole fraction of component i in the environment. Moreover, the exergy of fuel can be calculated using the following equation^34^:

6 $$\:\varepsilon\:=\frac{{ex}^{fuel}}{LHV}$$

With $$\:\varepsilon\:=\mathrm{1,06}$$^34^.

The applications of these equations to each piece of equipment in the studied layout are summarized in Table 4.

**Table 4:** Energy equations of the studied plant.

Component	Energy and exergy relations
Compressor	$\:{\dot{W}}_{comp}\:={\dot{m}}_{a}\times\:({h}_{b}-{h}_{a})$ $\:{\dot{Ė}x}_{destroyed,comp}=\:{Ėx}_{a}-{\dot{E}x}_{b}+{\dot{W}}_{comp}$
Combustion chamber	$\:{\dot{Q}}_{CC}={\dot{m}}_{a}\times\:\left({h}_{c}-{h}_{b}\right)={\dot{m}}_{f}\times\:LHV\times\:{\eta\:}_{CC}$ $\:{\dot{\dot{E}}x}_{destroyed,CC}=\:\:{\dot{\dot{E}}x}_{b}+\:{\dot{\dot{E}}x}_{fuel}^{chem}-\:{\dot{E}x}_{c}$
Gas turbine	$\:{\dot{W}}_{GT}={\dot{m}}_{g}\times\:\left({h}_{c}-{h}_{d}\right)$ $\:{\dot{Ė}x}_{destroyed,GT}={\dot{Ė}x}_{c}-{\dot{W}}_{GT}-{\dot{E}x}_{d}$
Heat recovery steam generator (HRSG)	$\:{\dot{Q}}_{HRSG}={\dot{m}}_{g}\times\:\left({h}_{d}-{h}_{e}\right)={\dot{m}}_{steam}\times\:\left({h}_{1}-{h}_{17}\right)$ $\:{\dot{E}x}_{destroyed,HRSG}=\:{\dot{E}x}_{d}\:+{\dot{E}x}_{17}-\:{\dot{E}x}_{e}+\:{\dot{E}x}_{1}$
Reheater	$\:{\dot{Q}}_{Reh}={\dot{m}}_{steam}\times\:\left({h}_{4}-{h}_{3}\right)={\dot{m}}_{f}\times\:LHV*{\eta\:}_{CC}$ $\:{\dot{E}x}_{destroyed,Reh}={\dot{E}x}_{3}+{\dot{E}x}_{g,in}-{\dot{E}x}_{4}-{\dot{E}x}_{g,out}$
Steam turbines	$\:{\dot{W}}_{ST}={\dot{m}}_{steam}\times\:({h}_{in}-{h}_{out})$ $\:{\dot{E}x}_{destroyed,ST}={\dot{E}x}_{in}^{}-{\dot{E}x}_{out}^{}-{\dot{W}}_{TV}$
Pumps	$\:{\dot{W}}_{Pum}={\dot{m}}_{steam}\times\:({h}_{out}-{h}_{in})$ $\:{\dot{E}x}_{destroyed,pum}={\dot{E}x}_{in,pum}+{\dot{W}}_{Pum}-{\dot{E}x}_{out,pum}$
FWHs	$\:{\dot{Q}}_{FWH}={\dot{m}}_{p}\times\:\left({h}_{p,in}-{h}_{p,out}\right)={\dot{m}}_{s}\times\:\left({h}_{s,out}-{h}_{s,in}\right)$ $\:{\dot{E}x}_{destroyed,FWH}={\dot{E}x}_{p,in}+{\dot{E}x}_{s,in}-{\dot{E}x}_{p,out}-{\dot{E}x}_{s,out}$
Condenser	$\:{\dot{Q}}_{cond}={\dot{m}}_{steam}\times\:\left({h}_{5}-{h}_{6}\right)={\dot{m}}_{cw}\times\:\left({h}_{27}-{h}_{26}\right)$ $\:{\dot{E}x}_{destroyed,cond}={\dot{E}x}_{5}+{\dot{E}x}_{26}-{\dot{E}x}_{6}-{\dot{E}x}_{27}$
Deaerator	$\:{\dot{Q}}_{Des}={\dot{m}}_{p}\times\:\left({h}_{p,in}-{h}_{p,out}\right)={\dot{m}}_{s}\times\:\left({h}_{s,out}-{h}_{s,in}\right)$ $\:{\dot{E}x}_{destroyed,Des}=\:\left(\:\sum\:{\dot{Ex}}_{s,in}-{ex}_{out}\times\:\sum\:{\dot{m}}_{s\:}\right)-\:{\dot{m}}_{p}\times\:{ex}_{out}-{\dot{Ex}}_{p,in}$

#### Economic modelling

Economic analysis is a crucial factor in the feasibility study of any investment project and industrial installation. The goal of this analysis is to determine the economic viability of the studied NGFCPP. In this study, economic analysis is conducted based on four key aspects: initial investment cost $\:{CI}_{Tot}$ (€), operating cost $\:{CO}_{op}$ (€/year), annual revenue $\:AR$ (€/year), and net present value *NPV* (€).

- Initial investment cost (engineering, construction, and site-related costs) $\:{CI}_{Tot}$.

The total initial investment cost of the studied combined plant can be expressed in terms of equipment/individual component cost as follows:

7 $$\:{CI}_{Tot}=f\:{\left[\sum\:_{i=1}^{n}{n}_{i}{CI}_{i}\right]}_{ST}+\:{CI}_{GT,block}$$

where: $\:{CI}_{GT,block}$ defines the initial investment cost of the gas turbine block is given by the following equation^18^:

8 $$\:{CI}_{GT,block}=3800\left({{MW}_{GT,block}}^{\mathrm{0,754}}\right)$$

While *f* is the factor for direct auxiliary installation, instrumentation and control, engineering, and plant start-up, equal to 1.87^35^. $\:{n}_{i}$ and $\:{Cl}_{i}$ represent the number of considered equipment in the steam turbine block, and its initial investment cost which is given by^35^:

9 $$\:{CI}_{i}=\:a\:\left({{MW}_{ST}}^{b}\right)$$

The regression constants *a* and *b* are provided in Table 5.

**Table 5:** Constants for the initial cost estimation of each component in the ST block^35,36^.

Equipment	a	b
Turbine	633,000	0.398
HRSG	1,340,000	0.694
Condenser	398,000	0.333
Condensation extraction pump	9,000	0.4425
Feedwater pump	35,000	0.6107
Other pumps	28,000	0.5575
FWH	51,000	0.5129
Deaerator	17,100	0.5575
Generator	138,300	0.3139

- Operating cost (overhead cost) $\:{CO}_{op}$.

The total annual operating cost ($\:{CO}_{op}$ Є/year) includes: Operating and maintenance costs of the gas turbine block ($\:{CO}_{GT,block}$ Є/year), Labor cost ($\:{CO}_{Lab}$Є/year), Fuel purchase cost ($\:{CO}_{f}$ Є/year), Maintenance and repair cost of the ST block ($\:{CO}_{MR}$ Є/year), Insurance and general costs ($\:{CO}_{inscgen}$ Є/year). Its formula is presented as^35^:

10 $$\:{CO}_{op}={CO}_{GT,block}+\:{CO}_{Lab}+{CO}_{f}+{CO}_{MR}+{CO}_{inscgen}$$

The parameters presented in Eq. (10) are calculated as:

11 $$\:{CO}_{GT,block}=\:0.04{\times\:CI}_{GT,block}$$

12 $$\:{CO}_{Lab}={n}_{emp}{CP}_{emp}$$

where $\:{n}_{emp}$​ is the number of employees, and $\:{CP}_{emp}$​ is the average labor cost per employee.

13 $$\:{CO}_{f}={m}_{f}{C}_{f}$$

14 $$\:{CO}_{MR}=0.015{CI}_{Tot}$$

15 $$\:{CO}_{inscgen}=\:0.01{\:CI}_{Tot}$$

- Annual revenue.

The annual revenue from electricity production is calculated as:

16 $$\:AR={f}_{auxi}\:MW\:hr\:\:{C}_{EP}$$

where $\:{f}_{auxi}$​ accounts for the energy needs of auxiliary equipment, and $\:{C}_{EP}$ is the current market price of electricity, set at approximately 315 Є/MW.

All reference values of the collected data for the analysis are presented in Table 6. However, taxes and financial charges have been neglected in this study.

**Table 6:** Data collected from the literature and records of an existing power plant^35–37^.

Parameter	Unit
Number of employees (–)	233
Average labor cost (Є)	3 000
Fuel cost (Є/kg)	1.28
Electricity cost $\:{\:{C}}_{{E}{P}}\mathrm{\:(}\mathrm{Є}\mathrm{\:/MW}$)	315
f Factor (-)	1.87
Operating hours per year hr (hrs/yr)	8000
Net power plant efficiency $\:{{f}}_{{a}{u}{x}{i}}$ (%)	90

- Net present value.

The final and most crucial parameter in this economic analysis is the net present value (NPV), which is calculated as:

17 $$\:NPV={\frac{(AR-{CO}_{op})}{{(1+D)}^{N}}}^{N}-\:{CI}_{Tot}$$

$$\:\mathrm{W}\mathrm{i}\mathrm{t}\mathrm{h}:\:D\:=\:0.09\:\mathrm{a}\mathrm{n}\mathrm{d}\:N=\:0:1:30\:\left(\mathrm{y}\mathrm{e}\mathrm{a}\mathrm{r}\mathrm{s}\right).$$

#### Environmental modelling

In this study, the environmental analysis focuses on the two most significant effects of using thermal power plants to generate electricity: CO_2_ emissions and water consumption for cooling. First, the CO_2_ emissions calculation is based on the stoichiometric combustion of natural gas (Methane). While for calculating the mass flow rate of cooling water, the general equation for energy balance equation across the condenser is applied:

18 $$\:{\dot{m}}_{cw}=\frac{{\dot{m}}_{steam}\times\:\left({h}_{5}-{h}_{6}\right)}{\left({h}_{27}-{h}_{26}\right)}$$

## Results and discussion

### Validation

**Fig. 2:**
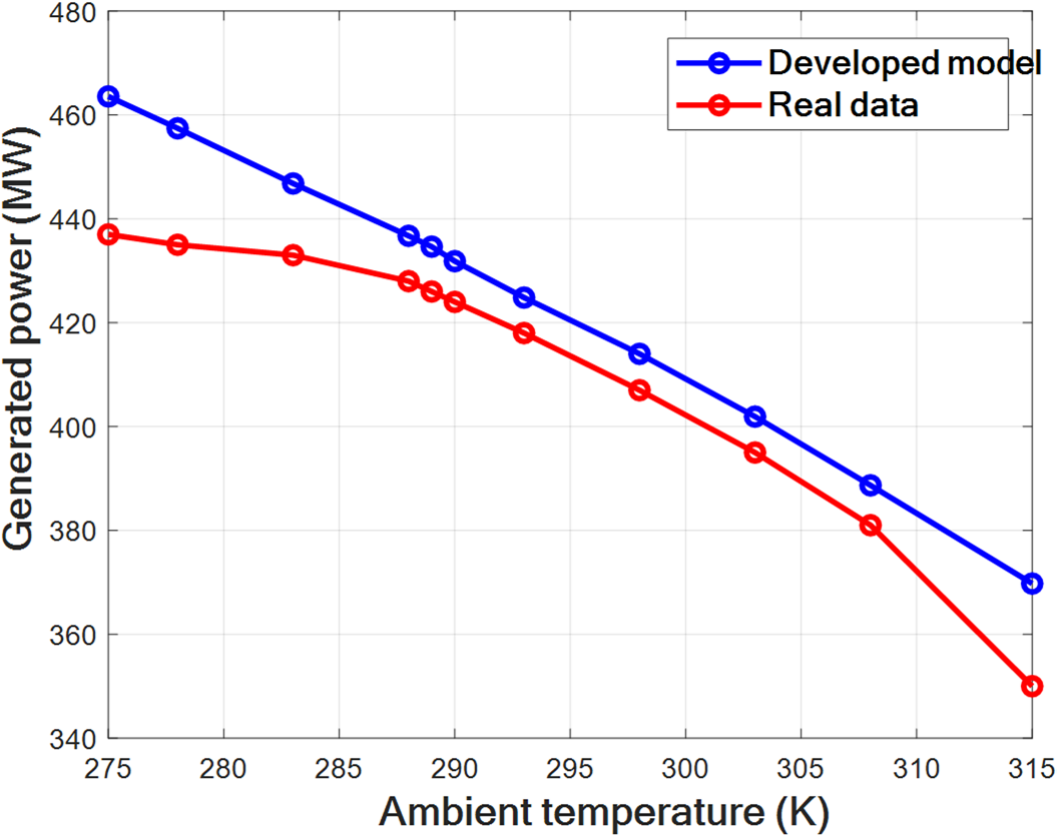
Validation of the developed model with real data.

The performances of the developed model are evaluated to confirm its viability. The results obtained by this model were compared with the real operating data of Hadjret Enouss power plant, where the validation process involved comparing the model’s predicted generated power under varying ambient temperatures with the real data of Hadjret Enouss power plant. For this purpose, four statistical parameters were used: the coefficient of determination (R²), the mean percentage error (MPE), the root mean square error (RMSE), and the coefficient of variance (COV). The results of this comparison between the developed energy model and the real operational data are summarized in Table 7 and visualized in Fig. 2.

**Table 7:** Validation of simulation results with real data.

Parameter	*R* ^2^	MPE (%)	RMSE (MW)	COV (%)
Value	0.9380	12.4758	13.6082	3.2102

The results of validation, summarized and presented in Table 7; Fig. 2, demonstrate a strong correlation with a high R^2^ of almost 0.94 between the developed model’s predictions and real operational data of the Hadjret Enouss power plant, and low RMSE and COV of less than 13.61 MW and 3.21% respectively. Consequently, the developed model presents a good correlation with the real data, confirming that the accuracy of the developed model is acceptable. However, the MPE reaches 12.48%, which is higher than the other statistical indicators and should be explicitly acknowledged as a limitation of the present modeling approach. This relatively high value of MPE suggests that while the model provides reliable trend prediction and acceptable average accuracy, noticeable deviations may occur under certain operating conditions. These discrepancies between the model and real data are primarily attributed to approximations and assumptions considered in the calculations. These include simplifying assumptions regarding constant isentropic coefficients, thermophysical properties, and limited access to detailed manufacturer data for certain components like those of different stages of turbines.

Furthermore, to validate the results of the ST block, the performances of the developed model were evaluated by comparing its outputs with the design specifications provided by the manufacturer of the Achouat operating steam power plant. For this validation, the relative error at selected points within the ST block was calculated and presented in Table 8.

**Table 8:** Statistical comparison between the results of our model and the manufacturer’s data.

Point	Parameter	Manufacturer data	Model	Error e (%)
Boiler inlet	Temperature (°C)	244	242.88	0.46
	Pressure (bar)	178.5	179.2	0.39
	Steam mass flow rate (kg/s)	171.5	169.33	1.28
HPT outlet	Temperature (°C)	329	316.29	4.01
	Pressure (bar)	26.70	26.70	0
	Steam mass flow rate (kg/s)	160.27	163.64	2.05
IPT inlet	Temperature (°C)	540	540	0
	Pressure (bar)	23.48	23.48	0
	Steam mass flow rate (kg/s)	148.22	149.49	0.84
Condenser outlet	Temperature (°C)	33.5	33.81	0.91
	Pressure (bar)	0.0527	0.0527	0
	Steam mass flow rate (kg/s)	125.25	143.29	12.58

After calculating the errors at certain key points between the modeled plant and the manufacturer’s data, a slight discrepancy was observed between the values estimated by the manufacturer and those obtained in this work. The error in steam mass flow rates ranged from a minimum of 0.84% at the IPT inlet to a maximum of 12.58% at the condenser outlet. The pressure error ranged from a minimum of 0% at most key points to a maximum of 0.39% at the boiler inlet. The temperature error ranged from a minimum of 0% at the IPT inlet to a maximum of 4.01% at the high-pressure turbine outlet. These discrepancies can be attributed to: (i) the approximations used to calculate isentropic compression and expansion coefficients, (ii) the approach used to estimate the thermophysical properties of water/steam, and (iii) the lack of manufacturer values such as the isentropic and mechanical efficiencies of some components.

Despite these errors, the model demonstrates a good correlation with the manufacturer’s data, confirming the viability of the established model.

###  4E comparative study

In this part of the present study, a comparison between three different configurations is presented: the first configuration is the Achouat power plant in Jijel, which operates on steam turbine cycle. The second configuration is the Hadjret Enouss power plant, which operates according to a reheat combined cycle. The last configuration is the new developed layout, which is based on a combined cycle with regeneration process in the ST block. For a meaningful and consistent comparison, the three analyzed power plant configurations were evaluated under the same boundary and operating conditions. Specifically, the same net power output, ambient temperature, fuel composition and lower heating value, pressure losses, and component performance assumptions were applied. Indeed, this comparison was conducted for an ambient temperature of 25 °C and a net power output of 407.56 MW fixed for the three configurations. The obtained results are summarized in Table 9.

**Table 9:** Comparative 4E study of the three configurations.

Parameter	Achouat steam power plant	Hadjret enouss power plant	Developed power plant
Generated power (MW)	407.56	407.56	407.56
Energy efficiency (%)	43.74	58.87	63.77
Exergy efficiency (%)	41.26	55.54	56.58
Fuel flow rate (kg/s)	90.17	15 0.14	14.86
CO_2_ emissions (kg/s)	88.34	41.54	40.77
Cooling water consumption (m^3^/s)	13.979	6.308	5.984
Investment cost (M€)	202.27	146.63	154.67
Operating cost (M€/year)	5.75	5.02	5.23
Annual revenue (M€/year)	92.43	92.43	92.43
NPV (M€/30 years)	702.0	765.1	756.5

According to Table 9, it can be seen that the third configuration (which presents the developed layout) has the highest energy and exergy efficiencies, with values of 63.77% and 56.58% respectively. This represents an energy and exergy gain of 0.45.79% and 37.13% respectively compared to the Achouat steam power plant, and 8.32% and 1.87% compared to the Hadjret Enouss power plant. This demonstrates, on one hand, the significant advantage of combined cycle plants over steam plants, and on the other hand, the indispensable role of regeneration systems in improving the performances and yield of power plant installations (whether steam or combined). This explains the superior energy and exergy efficiencies of the developed configuration over the conventional Hadjret Enouss configuration.

From an environmental perspective, the third configuration is stilling the best, with a fuel consumption of 14.86 kg/s, which is a reduction of 75.31 kg/s compared to the first configuration and 0.28 kg/s compared to the second. Additionally, this third configuration has the lowest CO_2_ emission rate at 40.7 kg/s, with a difference of 47.57 kg/s compared to the first configuration and 0.77 kg/s compared to the second. The high CO_2_ emissions in the first configuration (steam power plant) can be explained by its low efficiency compared to combined cycle plants, which requires more fuel to produce the same power as the other two configurations. Moreover, the developed plant emits less CO_2_ than Hadjret Enouss due to the preheating system, which reduces the amount of heat needed in the HRSG to reach the required steam temperature. This directly results in a reduction in fuel flow and CO_2_ emissions. Furthermore, the third configuration remains the best in terms of cooling water consumption with the lowest value of 5.984 m³/s, saving 0.323 m³/s compared to the Hadjret Enouss power plant, and 7.994 m³/s compared to the Achouat power plant. The latter consumes a very high cooling water flow rate because the steam flow required to produce the assumed power is very high, necessitating a large amount of cooling water to condense it at the low-pressure turbine (LPT) outlet. The difference in cooling water flow between the Hadjret Enouss and the developed plant is due to the regeneration process, which decreases the steam flow at the LPT outlet of the developed plant, thus reducing the condenser’s thermal load and consequently the cooling water consumption.

Economically, the Hadjret Enouss configuration is the best with the highest net present value (NPV) of more than 765 M€, with a profit of 63.1 M€ and 8.6 M€ compared to the Achouat and the developed plant respectively, and with a lower investment and operating cost of 146.63 M€ and 5.02 M€ respectively. The difference in NPV between Achouat and Hadjret Enouss is due to the high operating power for a steam plant, which increases the investment cost due to larger component sizes, and the operating cost will also be higher. The investment cost, operating cost, and NPV for the Achouat plant are 202.27 M€, 5.75 M€, and 713.64 M€ respectively. Regarding the difference between Hadjret Enouss and the developed plant, it is justified by the notable increase in investment cost when adding preheaters, which also increases the operating cost, thus reducing the NPV. The investment cost, operating cost, and NPV for the developed plant are 154.67 M€, 5.23 M€, and 764.57 M€ respectively. The annual electricity revenues are fixed for all configurations at 92.43 M€/year, as they have the same generated power.

After comparing the 4E performances of the three configurations, it can be said that while the Hadjret Enouss power plant is economically superior with the highest NPV compared to the other configurations, the developed plant is better from a sustainability perspective. It has the highest energy and exergy efficiencies compared to Hadjret Enouss, and the same is true for cooling water consumption. Indeed, 154.526 million kg of natural gas can be saved over the 35 years of operation of the developed plant compared to conventional NGFCPP, which implies a reduction in CO_2_ emissions of about 24.28 million kg/year. This preservation of one of the country’s strategic resources benefits future generations and helps protect the environment by reducing the greenhouse effect.

### Analysis of the 4E performances of the developed plant

As illustrated in subsection 3.2, the best configuration is the new layout proposed in the present work. Therefore, a detailed analysis of the 4E performances of this configuration is presented in the following subsections.

#### Energy analysis

**Fig. 3:**
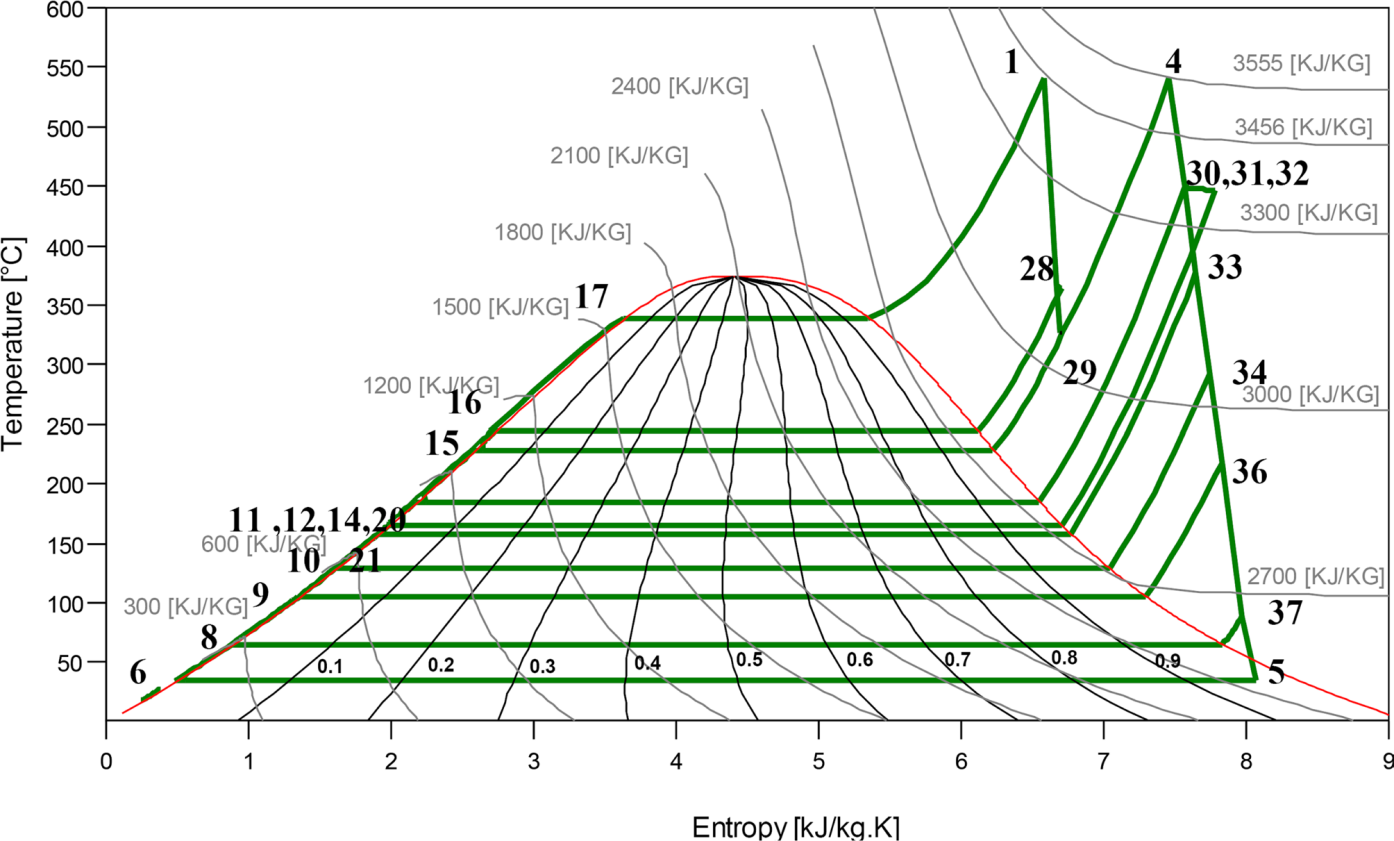
T-s. Diagram of the seven-extraction steam turbine.

**Fig. 4:**
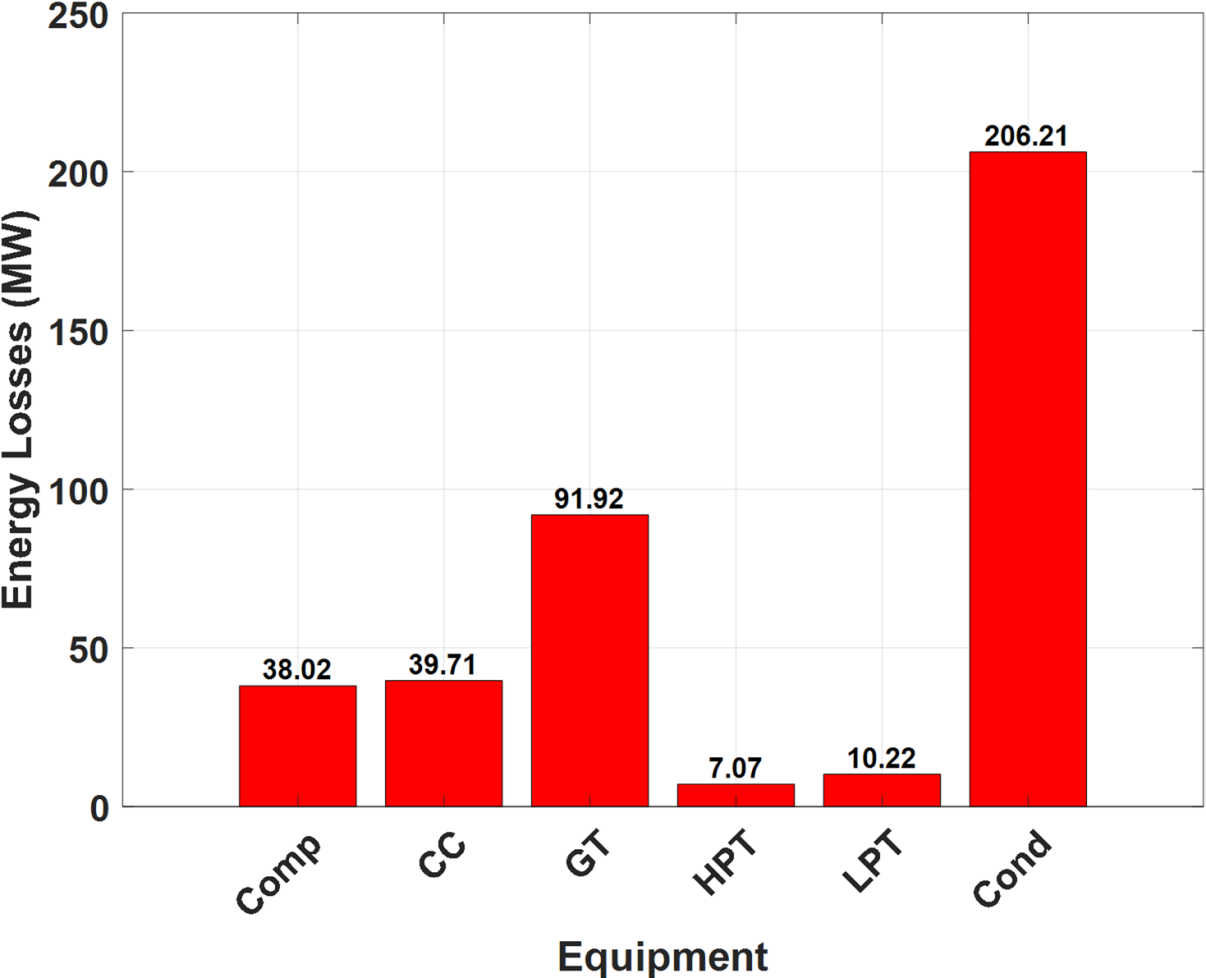
Energy losses in the main components of the installation.

A comprehensive summary of the thermodynamic state variables at various points in the developed layout of the studied NGFCPP was given in Table 10. These variables include temperature (T), pressure (P), enthalpy (h), entropy (s), mass flow rate (ṁ), and energy flux (Ė). These data are crucial for understanding the energy transformations and efficiency of the investigated power plant. Furthermore, Figs. 3 and 4 represent the T-s diagram of the steam/water in the ST block and the energy losses in the main components of the studied installation.

**Table 10:** Thermodynamic variables state at each point of the investigated plant.

Point	*P* (bar)	T (°C)	h (kJ/kg)	s (kJ/kg.K)	ṁ (kg/s)	Ė (MW)
a	0.9947	25	298.39	1.6951	603.33	180.02
b	18.20	472.26	762.22	2.6400	603.33	459.87
c	18.20	1396	1841.3	3.5752	618.47	1138.78
d	0.9947	642.02	951.20	2 0.8683	618.47	588.28
e	0.9947	82	355.77	1.8710	618.47	220.03
1	127.3	562	3505.4	6.6587	126.68	444.06
2	26.43	332.85	3084	6.7663	122.55	377.94
3	26.7	334.27	3095.1	6.7619	112.20	347.27
4	23.21	565	3609.5	7.5413	112.20	404.98
5	0.0621	36.78	2429.2	7.9596	88.48	214.93
6	0.0621	36.78	154.12	0.5294	107.79	16.61
7	24.54	36.95	156.95	0 0.5307	107.79	16.91
8	24.24	61.14	257.96	0.8443	107.79	27.80
9	23.64	101.05	425.24	1.3171	107.79	45.83
10	23.34	124.93	526.24	1.5790	107.79	56.72
11	22.74	154.44	652.52	1.8852	107.79	70.33
13	7.6110	168.35	712.01	2.0257	126.68	90.19
14	235.2	172.12	741.11	2.0347	126.68	93.19
15	232.8	183.66	790.79	2.1455	126.68	100.17
16	230.4	227.48	483.92	2.5495	126.68	61.30
17	228.1	242.80	1054.1	2.6880	126.68	133.53
18	35.2	234.48	1011.4	2.6501	4.13	4.17
19	26.7	190.66	811.16	2.2402	14.48	11.74
20	10.9	179.12	759.37	2.1311	16.77	12.73
21	5.79	129.93	546.32	1.6338	5.10	2.78
22	2.54	106.05	444.76	1.3749	9.12	4.05
23	1.17	661,400	276.95	0.9077	15.49	4.28
24	0.2410	41.950	175.73	0.5987	19.31	3.39
25	1.00	18.00	75.64	0.2682	6096.9	461.16
26	3.00	18.03	75.88	0.2683	6096.9	462.63
27	3.00	26.00	109.29	0.3815	6096.9	666.33
28	35.2	350.00	3162.3	6 0.7481	4.13	13.06
29	26.7	334.27	3095.1	6.7619	10.35	32.03
30	10.9	455.07	3380.9	7.5939	4.40	14.87
31	8.211	453.46	3380.9	7.7234	2.10	7.09
32	10.9	437.4	3380.9	7.5939	2 0.19	7.74
33	5.79	358.96	3212.5	7.6375	5.10	16.38
34	2.54	260.55	3022.3	7.6957	4.02	12.14
36	1.17	184.46	2869.2	7.7503	6.36	18.24
37	0.241	64.14	2625.2	7.8698	3.82	10.02

Notably, as shown in Table 10; Figs. 3 and 4, the combustion chamber outlet (point c) exhibits the highest temperature at 1396 °C, accompanied by a substantial enthalpy of 1841.3 kJ/kg, highlighting the intense energy exchange occurring at this stage. This point also has the largest energy flux of 1138.78 MW, representing the energy gained from burning the natural gas with air, underscoring its critical role in the overall energy conversion process.

From this energy flux, the gas turbine (GT) block generates a net power output of approximately 255 MW, with 368 MW lost through the flue gas at the turbine outlet. A significant portion of this lost heat, nearly 370 MW, is recovered by the heat recovery steam generator (HRSG) of the steam turbine (ST) block, which in turn produces a net power output of 160 MW. Conversely, substantial energy losses are evident at the condenser, accounting for 52% of the total energy losses within the entire installation (which represents an amount of more than 206 MW). The significant temperature and pressure drops recorded between points 5 and 6 highlight potential areas for efficiency improvements. This drop primarily stems from the heat (latent heat) loss required for the phase change of the working steam within the installation (from wet steam to liquid).

#### Exergy analysis

**Fig. 5:**
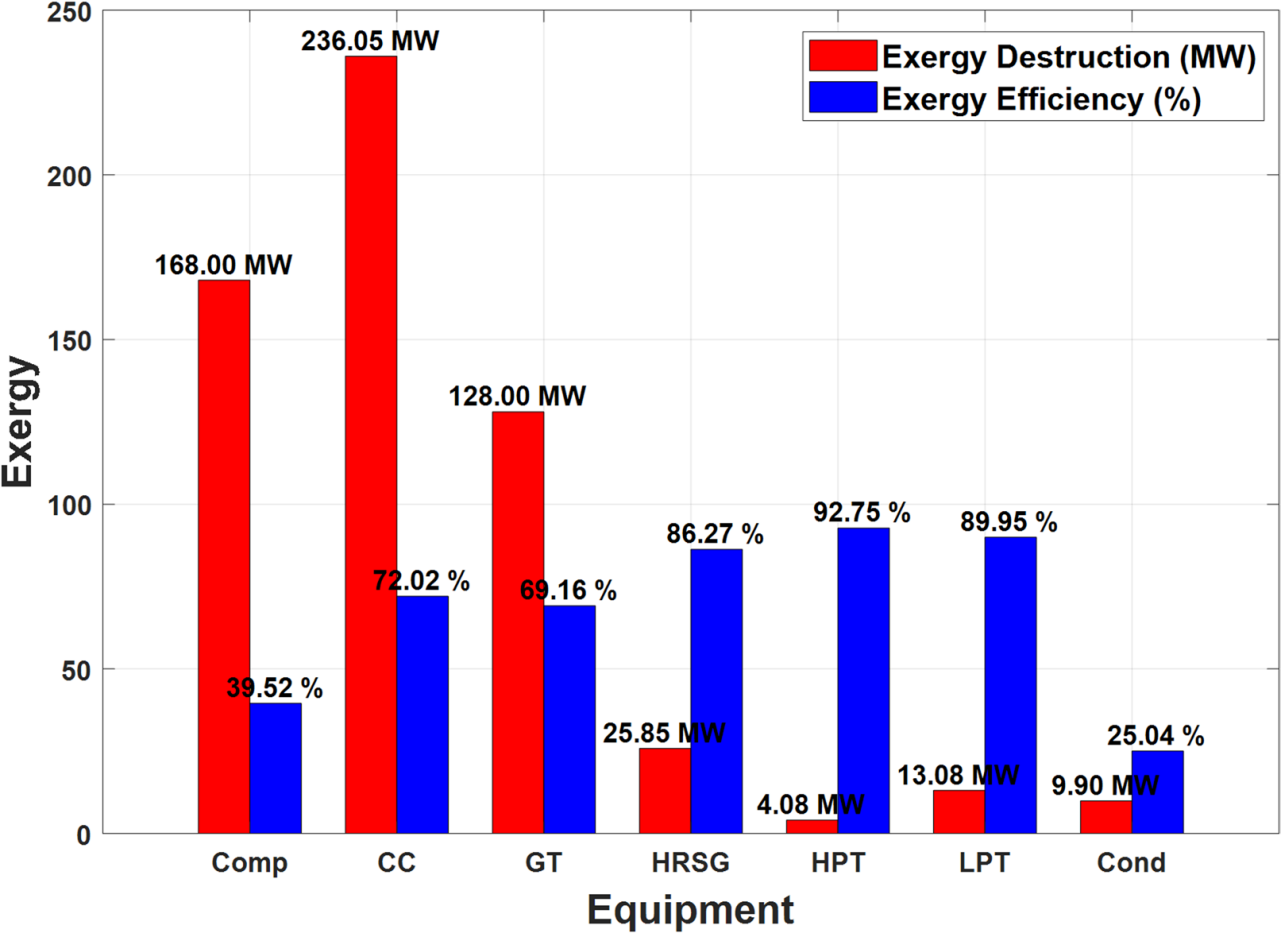
Destroyed exergy and exergy efficiency in the main components of the installation.

This section presents and discusses the exergy destruction and exergy efficiencies of the key components in the installation, as visualized in Fig. 5.

According to Fig. 5, the highest amounts of destroyed exergies are observed in the gas turbine section, while the steam turbine components represent the lowest sources of destroyed exergies in the installation. Moreover, the analysis of the developed NGFCPP shows that the combustion chamber has the highest exergy destruction at 236.05 MW, with a relatively high exergy efficiency of 72.02% which can be justified by the chemical reactions occur there, and significant temperature differences. Moreover, the compressor exhibits significant exergy losses with an exergy destruction of 168.00 MW and a lower efficiency of 39.52% due to the friction between the air getting to this component and its blades. The gas turbine, while more efficient than the compressor, still experiences considerable losses with 128.00 MW exergy destruction and 69.16% efficiency. The HRSG is notably efficient with an exergy destruction of 25.85 MW and an efficiency of 86.27%. The high-pressure turbine is the most efficient component, having the lowest exergy destruction at 4.08 MW and the highest efficiency at 92.75%. The low-pressure turbine also performs well with 13.08 MW exergy destruction and 89.95% efficiency. Finally, the condenser, as expected, shows the lowest efficiency at 25.04% and an exergy destruction of 9.90 MW due to significant heat rejection losses.

#### Economic analysis

**Fig. 6:**
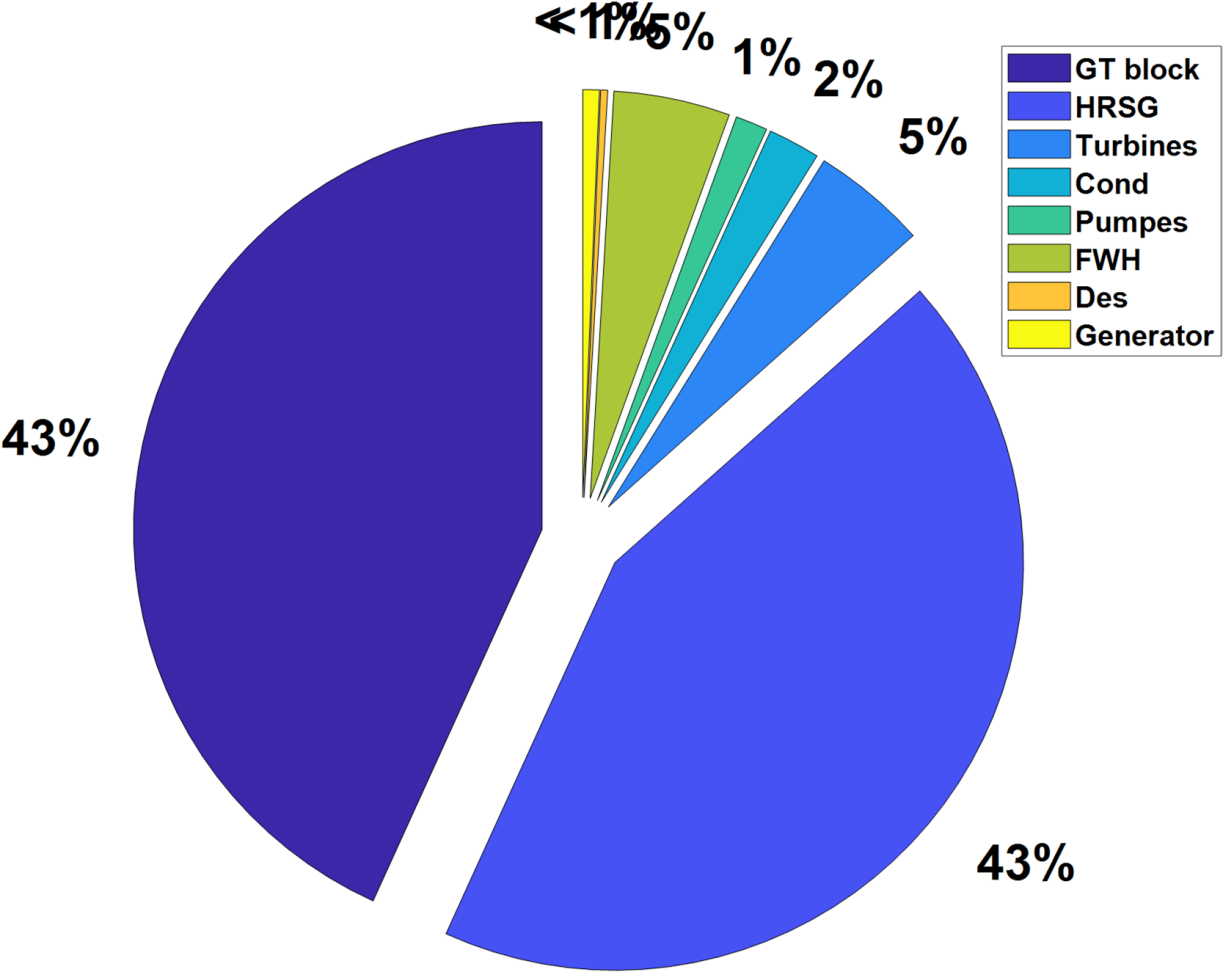
Investment cost of each component of the installation.

The first economic metric under consideration is the investment cost of the essential components of the studied plant, as depicted in Fig. 6.

Figure 6 shows the investment costs of the main components of the presented plant. The Gas Turbine (GT) block and the Heat Recovery Steam Generator (HRSG) each represent 43% of the total investment, equivalent to 45.28 M€ and 45.45 M€, respectively. This significant investment reflects their crucial roles in energy conversion and heat recovery. Turbines account for 5% (4.77 M€), necessary for converting thermal to mechanical energy, while the condenser, at 2% (2.16 M€), is essential for steam condensation. Pumps and feedwater heaters, at 1% (1.33 M€) and 5% (4.82 M€) respectively, are important for maintaining water/steam flow and preheating, enhancing thermal efficiency. The deaerator and generator, each less than 1% (0.29 M€ and 0.68 M€), are critical for fine-tuning steam properties and converting mechanical to electrical energy, despite their lower cost shares. The high costs of the GT block and HRSG are motivated by their essential roles in improving plant efficiency and reliability, while the lower costs of components like the deaerator reflect their smaller but still necessary contributions to the overall system performances.

**Fig. 7:**
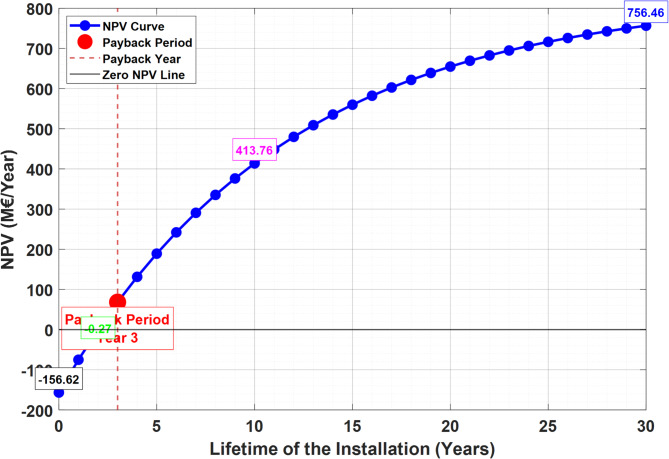
Variation of the NPV with the Plant’s lifetime.

The second economic metric, net present value (NPV), and its evolution over the plant’s 35-year operating lifetime are presented in Fig. 7.

The net present value (NPV) metric depicted in Fig. 7 reflects the financial viability of the plant over a 30-year period. Initially, the NPV is -156.62 M€, indicating substantial investment costs required for erecting the installation. As the plant becomes operational, annual operation and maintenance costs impact the NPV, yet over time, the annual revenue generated significantly surpasses these expenditures. By the 30th year, the NPV reaches 756.5 M€, demonstrating profitable returns. Furthermore, it is notable that the project begins to yield profits after two years of operation, the period necessary to recover the investment cost.

#### Environmental analysis

In the environmental study, the flow rate of air and the flow rate of cooling water required to burn 1 kg/s of fuel, as well as the rates of gases emitted into the atmosphere (carbon dioxide (CO_2_), water vapor (H_2_O), and nitrogen (N_2_)), will be calculated. According to the stoichiometric chemical reaction of the combustion of 1 kg/s of methane (CH_4_), the results are presented in the table below (Table 11).

**Table 11:** Results of the environmental analysis.

Parameter	Value
Airflow rate (kg/s)	20.26
CO_2_ emissions (kg/s)	2.754
H_2_O emissions (kg/s)	2.26
N_2_ emissions (kg/s)	13.184
Cooling water consumption (kg/s)	404.22
Specific CO_2_ emissions (kg/MWh)	24.3
Specific cooling water consumption (m^3^/MWh)	3.57

From Table 11, it can be concluded that the combustion of 1 kg/s of fuel (methane) in the plant requires an airflow rate of 20.26 kg/s and a cooling water mass of 404.22 kg/s. Additionally, 2.754 kg/s of carbon dioxide (CO_2_), 2.26 kg/s of water vapor (H_2_O), and 13.184 kg/s of nitrogen (N_2_) are emitted. For a net power output of 407.56 MW, these values correspond to a specific CO_2_ emission of approximately 24.33 kg/MWh and a specific cooling water consumption of about 3.57 m³/MWh, assuming 8000 operating hours per year.

## Conclusion

In this work, a novel design for a natural gas-fired combined power plant (NGFCPP) is presented and its 4E (energy, exergy, economic, and environmental) performances are assessed. The results are compared to those of a conventional operating power plant. Additionally, a comprehensive 4E analysis is conducted to gain a deeper understanding of the plant’s overall performances and yield. The main findings of this study are summarized below:

- The model demonstrated a strong correlation with real data from the Hadjret Enouss power plant, with an R² value of 0.9380. The mean percentage error (MPE) was 12.48%, and the root mean square error (RMSE) was 13.61 MW, indicating acceptable reliability despite some discrepancies due to approximations in calculations.
- The developed plant exhibited the highest energy efficiency at 63.77%, compared to 43.74% for the Achouat steam power plant and 58.87% for the Hadjret Enouss plant. This shows a significant improvement due to the regeneration process in the steam turbine block. On the other hand, the exergy efficiency of the developed plant was 56.58%, surpassing the Achouat plant’s 41.26% and the Hadjret Enouss plant’s 55.54%.
- The Hadjret Enouss plant had the highest net present value (NPV) of 776 M€. The developed plant had an NPV of 764.57 M€, reflecting a trade-off between higher investment costs and improved performances.
- The developed plant achieved the lowest CO_2_ emissions at 40.77 kg/s and the least cooling water consumption at 5.984 m³/s, highlighting its superior sustainability compared to the other configurations.
- The highest energy losses were observed at the condenser, accounting for 52% of total energy losses (over 206 MW). While the combustion chamber had the highest working temperature (1396 °C) and energy flux (1138.78 MW). Furthermore, the highest exergy destruction occurred in the combustion chamber (236.05 MW) with an efficiency of 72.02%. The compressor and gas turbine also showed significant exergy losses at 168.00 MW (39.52% efficiency) and 128.00 MW (69.16% efficiency), respectively.
- The total investment cost for the developed plant was 154.67 M€, with an annual operating cost of 5.23 M€. The NPV over 35 years was calculated to be 764.57 M€. Moreover, the developed plant reduced CO_2_ emissions by approximately 24.28 million kg/year and saved 154.526 million kg of natural gas over 35 years. The cooling water consumption was the lowest among the compared configurations, reinforcing its environmental benefits.

In conclusion, while the Hadjret Enouss plant offers the best economic performances, the developed combined cycle power plant excels in energy efficiency, exergy efficiency, and environmental sustainability, making it a superior choice for future power generation needs.

Several limitations in the present study can be identified, particularly regarding the accuracy of the developed model in areas where large local deviations were observed. These deviations primarily arise from considered assumptions, uncertainties in components modeling, and a lack of detailed manufacturer data. For example, the estimation of the mass flow rate at the condenser outlet contributes only marginally to global energy production, exergy destruction, and economic indicators; however, it influences the predicted water consumption of the studied plants.

Consequently, while locally noticeable, this deviation does not affect the main conclusions of the study, which focus on a comparative system-level performance analysis between the proposed layout and the alternative configurations. Therefore, future work should focus on extending the mathematical model to include off-design simulations, which would further reduce prediction errors and improve the accuracy of performance assessments.

Moreover, the influence of various operating and design parameters including compression ratio, excess air in combustion, as well as those related to the economic and environmental dimensions such as electricity price, discount rate, fuel cost, inlet cooling water temperature Should be thoroughly analyzed and discussed to provide a more comprehensive understanding of the plant’s behavior. In addition, the application of metaheuristic optimization techniques—such as genetic algorithms or even multi-objective optimization frameworks—is strongly recommended to refine operating conditions and further enhance the plant’s overall 4E performance.

### Annex

The validation of the developed model to simulate the energy performances of the studied combined power plant is presented in terms of five statistical parameters including relative error (e), Means Bias Error (MBE), Mean Percentage Error (MPE), Root Mean Square Error (RMSE), and Coefficient of correlation (R), which are written as follows:

19 $$\:e=\:\left(\frac{\left({x}_{est}-{x}_{man}\right)}{{x}_{man}}\right)$$

20 $$\:MBE=\:\frac{1}{n}\sum\:_{i=1}^{n}\left({x}_{est}-{x}_{man}\right)$$

21 $$\:MPE=\:\frac{100\%}{n}\sum\:_{i=1}^{n}\left(\frac{{x}_{man}-{x}_{est}}{{x}_{man}}\right)$$

22 $$\:RMSE=\:\sqrt{\sum\:_{i=1}^{n}\frac{{({x}_{est}-{x}_{man})}^{2}}{n}}$$

23 $$\:R=\:\frac{n\:\left(\sum\:{x}_{man}\:\times\:{x}_{est}\right)-(\sum\:{x}_{man})\times\:(\sum\:{x}_{est})}{\sqrt{\left[n\sum\:{x}_{man}^{2}-{(\sum\:{x}_{man})}^{2}\right]\times\:\left[\:n\sum\:{x}_{{x}_{est}}^{2}-{(\sum\:{x}_{est})}^{2}\right]}}$$

where: $\:n$, $\:{x}_{man}$, $\:{x}_{est}$ are the number of data points, the value provided by the manufacturer, and the model-estimated value respectively.

## Data Availability

The datasets used and/or analysed during the current study available from the corresponding author on reasonable request.

## List of symbols

*AR*: Annual revenue (€/year)
*C*_*EP*_: Current electricity price (€/MW)
*CI*: Initial investment cost (€)
*CO*: Cost (€/year)
*COV*: Coefficient of variance (%)
*D*: Discount rate (–)
$\dot{E}x$: Exergy flux (kW)
*h*: Enthalpy (kJ/kg)
*LHV*: Lower heating value (kJ/kg)
$\dot{m}$: Mass flow rate (kg/s)
*MPE*: Mean percentage error (%)
*N*: Year number (–)
*NPV*: Net present value (€)
*P*: Pressure (bar)
$\dot{Q}$: Heat flux (kW)
*R*^*2*^: Coefficient of determination (–)
*RMSE*: Root mean square error (MW)
*s*: Entropy (kJ/kg)
*T*: Temperature (K)
$$\dot{W}$$: Power (kW)

## Greek symbols

$\Delta P$: Pressure drop (bar)
$$\eta$$: Efficiency (–)

## Indices

0: Ambient/reference
chm: Chemical
cw: Cooling water
emp: Employee
f: Fuel
g: Gas
in: Inlet
Inscgen: Insurance and general
Lab: Labor
MR: Maintenance and repair
op: Operating
out: Outlet
p: Primary
ph: Physical
s: Secondary
steam: Steam
Tot: Total

## Abbreviations

4E: Energy, exergy, economic, and environmental
CC: Combustion chamber
CCGT: Combined cycle gas turbine
COMP: Compressor
COND: Condenser
DES: Deaerator
ECO: Economizer
EVAP: Evaporator
FWH: Feedwater heater
GEN: Generator
GT: Gas turbine
HPT: High-pressure turbine
HRSG: Heat recovery steam generator
LPT: Low-pressure turbine
NGFCPP: Natural gas-fired combined power plant
PUM: Pump
REH: Reheater
ST: Steam turbine
SUP: Superheater
